# Serelaxin Elicits Bronchodilation and Enhances β-Adrenoceptor-Mediated Airway Relaxation

**DOI:** 10.3389/fphar.2016.00406

**Published:** 2016-10-27

**Authors:** Maggie Lam, Simon G. Royce, Chantal Donovan, Maria Jelinic, Laura J. Parry, Chrishan S. Samuel, Jane E. Bourke

**Affiliations:** ^1^Biomedicine Discovery Institute and Department of Pharmacology, Monash University, ClaytonVIC, Australia; ^2^Department of Pharmacology, Lung Health Research Centre, The University of Melbourne, ParkvilleVIC, Australia; ^3^School of BioSciences, The University of Melbourne, ParkvilleVIC, Australia

**Keywords:** airway, β-adrenoceptor agonist, bronchodilation, epithelium, precision-cut lung slice, relaxin, rosiglitazone, trachea

## Abstract

Treatment with β-adrenoceptor agonists does not fully overcome the symptoms associated with severe asthma. Serelaxin elicits potent uterine and vascular relaxation via its cognate receptor, RXFP1, and nitric oxide (NO) signaling, and is being clinically evaluated for the treatment of acute heart failure. However, its direct bronchodilator efficacy has yet to be explored. Tracheal rings were prepared from male Sprague-Dawley rats (250–350 g) and tricolor guinea pigs, and precision cut lung slices (PCLSs) containing intrapulmonary airways were prepared from rats only. Recombinant human serelaxin (rhRLX) alone and in combination with rosiglitazone (PPARγ agonist; recently described as a novel dilator) or β-adrenoceptor agonists (isoprenaline, salbutamol) were added either to pre-contracted airways, or before contraction with methacholine or endothelin-1. Regulation of rhRLX responses by epithelial removal, indomethacin (cyclooxygenase inhibitor), L-NAME (nitric oxide synthase inhibitor), SQ22536 (adenylate cyclase inhibitor) and ODQ (guanylate cyclase inhibitor) were also evaluated. Immunohistochemistry was used to localize RXFP1 to airway epithelium and smooth muscle. rhRLX elicited relaxation in rat trachea and PCLS, more slowly than rosiglitazone or isoprenaline, but potentiated relaxation to both these dilators. It markedly increased β-adrenoceptor agonist potency in guinea pig trachea. rhRLX, rosiglitazone, and isoprenaline pretreatment also inhibited the development of rat tracheal contraction. Bronchoprotection by rhRLX increased with longer pre-incubation time, and was partially reduced by epithelial removal, indomethacin and/or L-NAME. SQ22536 and ODQ also partially inhibited rhRLX-mediated relaxation in both intact and epithelial-denuded trachea. RXFP1 expression in the airways was at higher levels in epithelium than smooth muscle. In summary, rhRLX elicits large and small airway relaxation via epithelial-dependent and -independent mechanisms, likely via RXFP1 activation and generation of NO, prostaglandins and cAMP/cGMP. rhRLX also enhanced responsiveness to other dilators, suggesting its potential as an alternative or add-on therapy for severe asthma.

## Introduction

Asthma is a chronic inflammatory disease of the airways, affecting 300 million world-wide (Masoli et al., 2004). Recruitment of inflammatory cells and stimulation of resident structural cells in asthma promotes epithelial damage, goblet cell metaplasia, fibrosis and the accumulation of airway smooth muscle (ASM) (Mauad et al., 2007). These inflammatory and structural changes contribute to the development of airway hyperresponsiveness (AHR), characterized by excessive bronchoconstriction to allergic and non-allergic stimuli (Yeganeh et al., 2013).

While treatment with the β_2_-adrenoceptor agonist salbutamol (SAL) generally reverses asthma symptoms, dilator responses in many patients may be limited by factors such as frequent use and/or viral infection leading to tolerance and receptor desensitization (Duechs et al., 2014; Spina, 2014). β_2_-adrenoceptor agonists have also been shown to have relatively lower efficacy in small airways in the distal lung, where increased inflammation and AHR contributes to asthma severity (Donovan et al., 2013; Bourke et al., 2014). Thus, it is important to identify novel agents to reverse or inhibit the development of contraction to act as bronchodilators or bronchoprotective agents in both large and small airways.

Serelaxin (rhRLX or RLX) is the recombinant drug-based version of the major stored and circulating form of the human gene-2 relaxin hormone, which mediates its physiological actions via activation of Relaxin Family Peptide Receptor 1 (RXFP1) (Samuel et al., 2007). Although rhRLX is generally associated with vascular changes in pregnancy and childbirth, its recent clinical assessment for acute heart failure was associated with beneficial effects on hemodynamics and reduced mortality (Teerlink et al., 2013). These findings were consistent with rhRLX-enhanced vasorelaxation in isolated rodent small renal and mesenteric arteries (McGuane et al., 2011; Leo et al., 2014) and human gluteal and subcutaneous resistance arteries (Fisher et al., 2002; McGuane et al., 2011). rhRLX has also been shown to influence gastrointestinal motility in mice via the generation of nitric oxide (NO) from an intact epithelial layer (Baccari et al., 2013).

Of relevance to asthma, chronic *in vivo* administration of rhRLX inhibited fibrosis and the development of AHR in a mouse model of ovalbumin-induced allergic airways disease (AAD) mimicking key features of asthma (Royce et al., 2009, 2013a). Although rhRLX is able to exert protective effects in the lung, and has also been identified as a safe and efficacious relaxant of vascular and uterine smooth muscle (Bani et al., 1998; Tan et al., 1998), its acute effects on airway contraction have yet to be characterized.

Another potential novel bronchodilator of interest is rosiglitazone (RGZ), originally identified as a potent agonist of peroxisome proliferator activated receptor γ (PPARγ). RGZ has recently been shown to elicit acute airway relaxation independently of PPARγ activation in mouse precision cut lung slices (PCLS) (Donovan et al., 2014) and mouse trachea (Donovan et al., 2015). Furthermore, RGZ was more efficacious than β_2_-adrenoceptor agonists in mouse airways, albeit at lower potency (Donovan et al., 2014). Like rhRLX, RGZ has been shown to inhibit fibrosis and the development of AHR in mouse models of allergic AAD (Honda et al., 2004; Ward et al., 2004; Donovan et al., 2012).

Given this background, the aims of this study were to assess the potential bronchodilator and bronchoprotective effects of rhRLX in comparison to RGZ and the β-adrenoceptor agonists salbutamol and isoprenaline (ISO). We hypothesized that rhRLX would both reverse established airway contraction and inhibit the development of contraction, with potential for additivity with these other dilators.

## Materials and Methods

### Materials

Acetylcholine (ACh), methacholine (MCh), substance P, isoprenaline (ISO), salbutamol (SAL), and *N*ω-nitro-L-arginine methyl ester (L-NAME) (all from Sigma-Aldrich, St. Louis, MO, USA); endothelin-1 (ET-1, GenScript, Piscatawa, NJ, USA); indomethacin and rosiglitazone (RGZ) (both from Cayman Chemical, Ann Arbor, MI, USA); recombinant human gene-2 relaxin (serelaxin/ rhRLX, kindly provided by Novartis AG, Basel, Switzerland).

### Animals and Tissue Collection

All procedures were approved by a Monash University Animal Ethics Committee (AEC number: MARP2/2014/066), which adheres to the Australia Code of Practice for the Care and Use of Animals for Scientific Purposes. Male Sprague-Dawley rats (250–350 g) (Monash University Animal Services, Melbourne, VIC, Australia) and Tricolor guinea pigs (8–10 weeks) (Pipers, Cowra, NSW, Australia) were housed in the Animal Facility, Monash University and maintained on a fixed 12 h light, 12 h dark lighting schedule, with free access to food and water at all times.

Rats were humanely euthanized by sodium pentobarbitone overdose (60 mg/mL administered i.p. at a volume of 1 mL per 100 g body weight) for preparation of tracheal tissue and PCLS. Guinea pigs were gently restrained and stunned by a single blow to the head. Loss of consciousness was confirmed by the lack of withdrawal reflex, followed by immediate exsanguination of the carotid artery.

### Myograph Experiments

Trachea were quickly dissected and mounted in Krebs-Henseleit (59 mM NaCl, 2.3 mM KCl, 0.69 mM MgSO_4_⋅7H_2_O, 2.5 mM CaCl_2_⋅6H_2_O, 0.6 mM KH_2_PO, 10 mM EDTA, 25 mM NaHCO_3_, and 6 mM glucose) buffer solution for experiments using myograph (Danish MyoTechnology, Arthuus Denmark, 5 ml bath for rat trachea) or standard organ baths (10 ml bath for guinea pig trachea). Tissues were set to optimum resting tension for maximum development of contraction to potassium physiological salt solution (KPSS; 123.7 mM K^+^). A maximal contraction to ACh (30 μM) was also obtained in all tissues. To assess airway relaxation, cumulative additions of MCh were made to establish a submaximal contraction (50–70% of ACh maximum). Concentration-response curves were then constructed to rhRLX, RGZ, ISO, or SAL or selected concentrations of rhRLX (0.1 μM), RGZ (10, 100 μM) or ISO (1, 10 μM) were added alone or in combination.

To assess potential inhibition of airway contraction, trachea were pre-incubated with selected concentrations of rhRLX (0.1 μM), RGZ (100 μM), or ISO (10 μM) for 30 min prior to the construction of cumulative MCh or ET-1 concentration-response curves. The contribution of epithelial-derived factors to inhibition of airway contraction was assessed in trachea pre-incubated in the absence or presence of rhRLX (0.1 μM) for 2 h. The bath solution was then either left or replaced before concentration-response curves were generated to ET-1. Alternatively, the epithelium was removed by gentle agitation of the lumen using a wooden toothpick and confirmed by loss of relaxation to the epithelial-dependent dilator substance P (1 μM) and H&E staining of tracheal sections after experiments. The contribution of NO and cyclooxygenase (COX)-derived mediators was assessed using the NO synthase (NOS) inhibitor, L-NAME (100 μM) and/or the COX inhibitor, indomethacin (3 μM) added during the rhRLX pre-incubation period before ET-1 curves.

Finally, SQ22536 (10 μM) and/or ODQ (1 μM), inhibitors of adenylate cyclase and guanylate cyclase respectively, were added to both intact and denuded trachea before a submaximal contraction to MCh was established for assessment of relaxation to rhRLX (0.1 μM).

### Precision Cut Lung Slice (PCLS) Experiments

Precision cut lung slice were prepared with minor modifications from previously published methods (Bourke et al., 2014; Donovan et al., 2014). Rats were euthanized as described previously and dissected to expose the heart and trachea. Heparin sodium (500IU) was injected into the left ventricle to empty the lungs of blood before cannulation. Trachea were cannulated with a catheter containing two ports (20 G Intima, Becton Dickinson, VIC, Australia) and lungs were inflated with ∼10 mL agarose gel (2% in 1x HBSS at 37°C), followed by a bolus of ∼3 mL air. Lungs were cooled by bathing in cold HBSS/HEPES and the rats were then kept at 4°C to allow the agarose to solidify, before the lungs were removed. The upper right lobe was isolated and adhered with cyanocrylate to a mounting plate in a vibratome (Compresstome, Precisionary Instruments, Greenville, NC, USA). PCLS of 200 μm thickness were prepared and transferred into cell culture plates containing DMEM, supplemented with 1% penicillin-streptomycin and incubated for 24 h (37°C, 5% CO_2_) prior to experiments.

Precision cut lung slice were transferred to HBSS/HEPES and mounted in custom-made perfusion chambers (∼100 μL volume). A viable airway (∼200 μm diameter) was selected from each slice based on the presence of an intact layer of epithelial cells with ciliary activity. PCLS were perfused at a constant rate (∼0.5 mL/min) through an eight-channel gravity-fed perfusion system under vacuum. Slices were initially perfused with MCh (0.3 μM) to establish a submaximal pre-contraction, then perfused with MCh in combination with rhRLX (0.1 μM) or ISO (10 μM). Perfusion was then stopped to permit assessment of airway responses to MCh in the presence of dilator under static conditions.

Phase contrast microscopy was conducted using an inverted microscope (Nikon Ti-U, Melville, NY, USA) to observe drug-induced airway changes, employing 10X objective lens, zoom adapter, reducing lens and camera (CCD camera model TM-62EX; Pulnix, Takex, Japan). Changes in airway lumen area were captured as digital images (744 × 572 pixels) in time lapse (0.5 Hz) using image acquisition software (Video Savant; IO Industries, Inc., London, ON, Canada). Obtained files were converted to TIFF files and analyzed using NIH/Scion software (Scion Corporation; download www.scioncorpcom).

### Immunohistochemistry

Lungs were dissected whole and fixed in neutral buffered formalin before being embedded in paraffin wax. Sections (5 μm) were cut and mounted on SuperFrost PLUS slides (Menzel-Gläser, Braunschweig, Germany). Procedures for bright field immunohistochemistry are detailed in (Jelinic et al., 2014). Lung sections were incubated overnight at 4°C with 3 μg/ml rat RXFP1 antiserum (#107, raised against amino acid residues 107–119 of the rat RXFP1 protein) or preimmune serum (rabbit IgG). Immunoreactivity was detected using the MACH 2^TM^ system (Biocare Medical, Concord, CA, USA) and 3, 3′ diaminobenzidine (DAB) as the chromagen substrate (Vector Laboratories, Burlingame, CA, USA). The rat RXFP1 antibody used has previously been shown to be selective, with no cross-reactivity to another relaxin-family peptide receptor (RXFP2) (Jelinic et al., 2014).

### Statistical Analysis

Contraction was normalized to the maximum ACh contraction for MCh or the KPSS response for ET-1 in trachea, or expressed as % initial airway area in PCLS. Relaxation was expressed as % of the MCh submaximal pre-contraction. All data are expressed as the mean ± SEM. Non-linear regression of concentration-response curves to obtain fitted maxima and pEC50 values where possible. Results were analyzed via *t*-test or one-way ANOVA for multiple comparisons between groups as appropriate; where statistical significance was accepted at *P* < 0.05. All data analysis was performed using GraphPad Prism v6 (GraphPad Software, San Diego, CA, USA).

## Results

### rhRLX, RGZ, and ISO Elicit Relaxation of Rat Trachea

**FIGURE 1:**
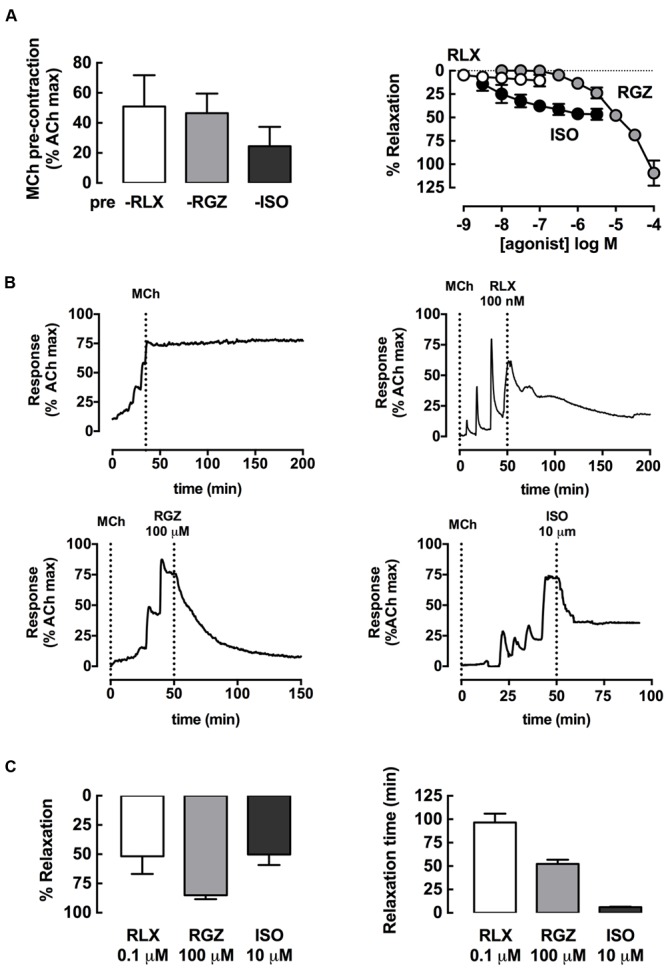
**rhRLX, RGZ, and ISO elicit relaxation of rat trachea.** Rat trachea were mounted under isometric conditions for optimum development of contraction. Trachea were **(A)** precontracted with MCh, prior to preparation of concentration-response curves to rhRLX (RLX), RGZ, or ISO. **(B)** Representative traces show relaxation to RLX, RGZ, and ISO after MCh, as well as a time control for MCh alone. **(C)** Relaxation responses and time to maximum relaxation to single additions of RLX (0.1 μM), RGZ (100 μM), or ISO (1 μM) were also assessed. Extent of relaxation is expressed as % of submaximal MCh pre-contraction, with relaxation time expressed in min (mean ± SEM for *n* = 4 per group).

Dilator effects of rhRLX, RGZ, and ISO in rat trachea were compared after pre-contraction to similar submaximal levels with MCh (300 nM), measuring changes in isometric force in a static organ bath (5 ml volume; **Figure 1A**). When added at 5–10 min intervals, rhRLX did not cause any relaxation up to 0.1 μM, the highest concentration available (**Figure 1B**). In contrast, RGZ and ISO induced concentration-dependent relaxation (fitted maximum % relaxation: ISO 45.6 ± 5.3; RGZ 138.1 ± 30.0; *P* < 0.05). When compared to RGZ, ISO was more potent (pEC_50_: ISO 8.0 ± 0.3; RGZ 4.7 ± 0.2; *P* < 0.001), but only RGZ elicited complete relaxation (**Figure 1A**).

When the highest concentrations of each drug tested were allowed to remain in contact with the pre-contracted trachea, marked relaxation to rhRLX, as well as to RGZ and ISO, became evident. Representative traces show the effects of addition of rhRLX (0.1 μM), RGZ (100 μM), and ISO (1 μM) relative to a time control showing maintained contraction to MCh alone over 200 min (**Figure 1B**). Partial relaxation to rhRLX occurred at slower rate than either RGZ or ISO, which caused near complete and partial tracheal relaxation within approximately 50 and 5 min respectively (**Figures 1B,C**). Notably, the extent of relaxation to 0.1 μM rhRLX-mediated (% relaxation: 52 ± 15%) was comparable to the maximal ISO-induced airway relaxation (50 ± 9%).

### rhRLX Potentiates Tracheal Relaxation to β-Adrenoceptor Agonists and RGZ

**FIGURE 2:**
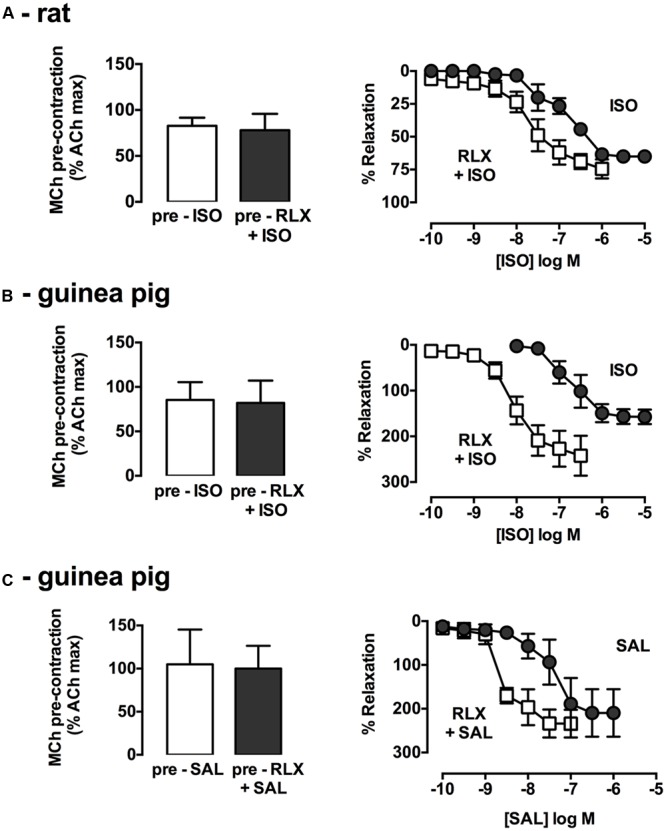
**rhRLX potentiates relaxation to β-adrenoceptor agonists.** Trachea were precontracted with MCh, prior to measurement of relaxation responses in the absence and presence of rhRLX (100 nM) to ISO (0.001–10 μM) in **(A)** rat and **(B)** guinea pig trachea or SAL (0.001–10 μM) in **(C)** guinea pig trachea. Extent of relaxation is expressed as % of submaximal MCh pre-contraction (mean ± SEM for *n* = 4–5 per group).

The combined effects of rhRLX and β-adrenoceptor agonists were explored. In rat trachea, where activation of both β_1_ and β_2_ adrenoceptors contributes to relaxation, ISO alone did not elicit complete relaxation, and the apparent leftward shift in its concentration-response in the presence of rhRLX did not reach significance (potency *P* = 0.11; efficacy *P* = 0.08, *n* = 4–6, **Figure 2A**).

As in rat trachea, rhRLX alone did not cause rapid relaxation of guinea pig trachea (data not shown). Consistent with the predominant role of β_2_-adrenoceptor in guinea pig airways, the selective β_2_-adrenoceptor agonist SAL was more potent than the β_1/_β_2_-adrenoceptor agonist ISO (pEC_50_: SAL 7.4 ± 0.3, ISO 6.6 ± 0.2, **Figures 2B,C**), with both dilators causing relaxation below the baseline prior to MCh. Pre-incubation with rhRLX resulted in a 27.1-fold increase in ISO potency (*P* < 0.001, *n* = 4, **Figure 2B**) with a similar leftward shift seen for SAL in the presence of rhRLX (*P* < 0.05, *n* = 3, **Figure 2C**).

**FIGURE 3:**
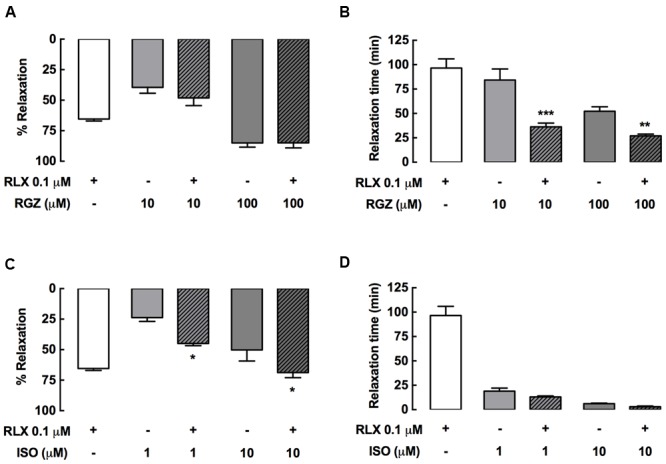
**rhRLX potentiates tracheal relaxation to RGZ and ISO.** Rat trachea were precontracted with MCh, prior to measurement of **(A,C)** relaxation responses and **(B,D)** time to relaxation for rhRLX (RLX, 0.1 μM), RGZ (10, 100 μM) and ISO (0.1, 1 μM) alone and in combination. Extent of relaxation is expressed as % of submaximal MCh pre-contraction, with relaxation time expressed in min (mean ± SEM for *n* = 4–5 per group). ^∗^*P* < 0.05, ^∗∗^*P* < 0.01, ^∗∗∗^*P* < 0.001 vs. the matched concentration of RGZ or ISO alone.

Effective single dilator concentrations of rhRLX, RGZ, and ISO were also tested in combination in rat trachea (**Figure 3**). Combining rhRLX (0.1 μM) with a submaximal concentration of RGZ (10 μM) did not induce greater relaxation compared to RGZ alone, while near-maximal relaxation to RGZ (100 μM) was maintained in the presence of rhRLX (**Figure 3A**). However, the addition of rhRLX markedly increased the rate of relaxation to both concentrations of RGZ tested (*P* < 0.001, *P* < 0.01 vs. RGZ alone, **Figure 3B**).

In contrast, the addition of rhRLX resulted in greater relaxation to ISO (0.1, 10 μM) (*P* < 0.05 vs. ISO alone), increasing the efficacy of both the submaximal and maximally effective concentrations of ISO (**Figure 3C**). The rapid rate of ISO-mediated relaxation was not further increased by rhRLX (**Figure 3D**).

### rhRLX Elicits Relaxation of Rat Intrapulmonary Airways in Lung Slices

**FIGURE 4:**
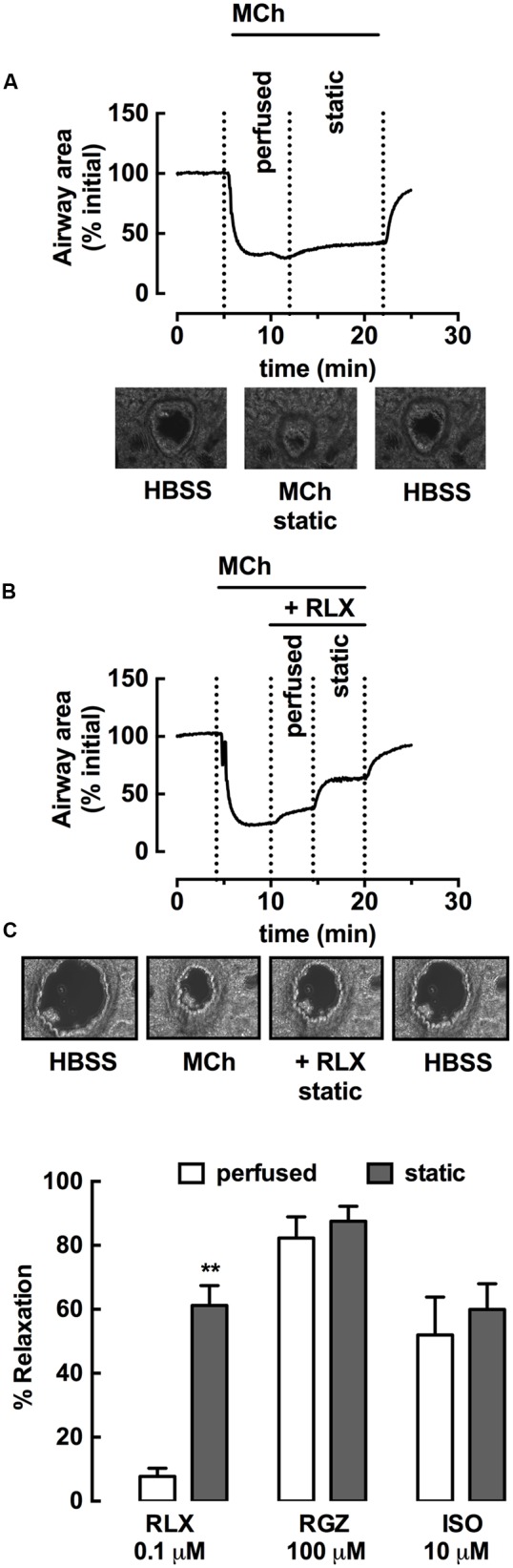
**rhRLX elicits relaxation of rat intrapulmonary airways in lung slices.** Rat lung slices were mounted in a customized chamber for assessment of changes in intrapulmonary airway area by phase-contrast microscopy. Airways were pre-contracted with MCh (0.3 μM) under perfused and static conditions, in the absence and presence of rhRLX (RLX, 0.1 μM). Representative time-course and phase-contrast images of the last frame of each condition for **(A)** MCh alone and **(B)** MCh followed by RLX (0.1 μM). Traces show area values in pixels determined from gray-scale analysis of images using VideoSavant. **(C)** Grouped data shows relaxation by RLX, RGZ (100 μM) and ISO (10 μM) under perfused and static conditions. Data is expressed as % relaxation of MCh pre-contraction (mean ± SEM, *n* = 4 per group). ^∗∗^*P* < 0.01 vs. relaxation to RLX under perfused conditions.

Using rat PCLS, the effect of rhRLX (0.1 μM) on intrapulmonary airway contraction was also assessed, measuring changes in airway area under perfused and static conditions in a small volume customized chamber (∼100 μl volume). A representative trace and sequential images of the same airway in PCLS show that the stable contraction to MCh during continuous perfusion over the lung slice was maintained under static conditions (**Figure 4A**). rhRLX rapidly and partially reversed the MCh-induced reduction in airway area (**Figure 4B**), with greater relaxation achieved under static than perfused conditions (% relaxation: perfused 7 ± 3%, static 64 ± 8%, *P* < 0.001; **Figure 4C**). In contrast, relaxation to both RGZ and ISO was similar under perfused and static conditions, causing near-complete and partial relaxation respectively, similar to that seen in trachea (**Figure 4C**).

### rhRLX and RGZ, but not ISO, Inhibit the Development of Tracheal Contraction

**FIGURE 5:**
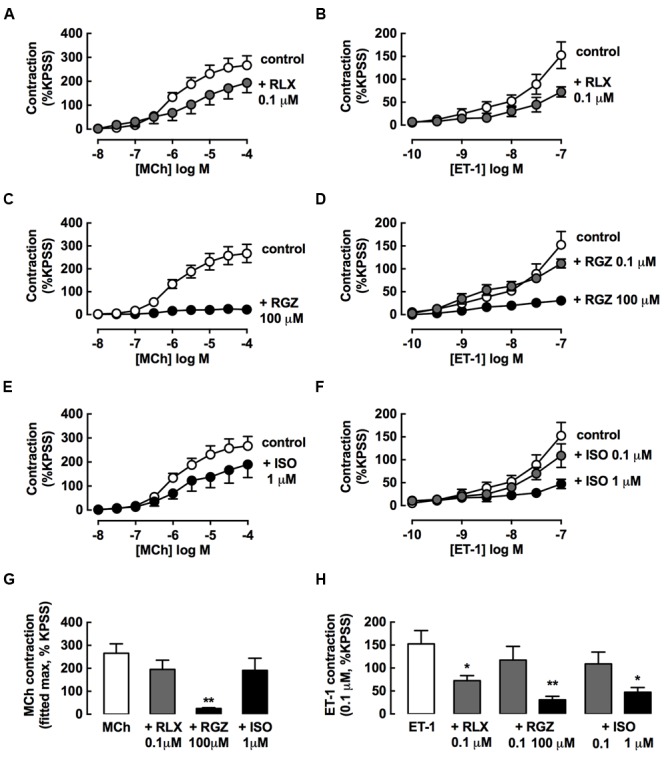
**rhRLX and RGZ, but not ISO, inhibit the development of tracheal contraction.** The effects of pretreatment with rhRLX (RLX), RGZ and ISO (30 min) on the development of contraction to MCh (left panels) and ET-1 (right panels) were assessed, testing **(A,E)** RLX (0.1 μM), **(B,F)** RGZ (0.1, 100 μM), and **(C,G)** ISO (0.1, 1 μM). For clarity, the same control data is shown on panels **(A–C)** for MCh and panels E-G for ET-1. **(D)** Maximum contraction to MCh determined from fitted concentration-responses curves using GraphPad Prism. **(H)** Contraction to the highest tested concentration of ET-1 (0.1 μM). All responses are expressed as % KPSS standard contraction (mean ± SEM, *n* = 4–5 per group). ^∗^*P* < 0.05, ^∗∗^*P* < 0.01 vs. MCh or ET-1 alone.

The bronchoprotective effects of rhRLX, RGZ, or ISO in inhibiting the development of rat tracheal contraction were then compared. Concentration-dependent contraction was established, with full and partial curves obtained for MCh and ET-1 respectively over the concentration ranges tested (**Figure 5**).

Pre-incubation with an effective dilator concentration of RGZ (100 μM) for 30 min inhibited the development of contractile responses to MCh by 90% (**Figure 5B**). Although, the maximum contraction to MCh was reduced by 25% with either rhRLX (0.1 μM) (**Figure 5A**) or ISO (10 μM) (**Figure 5C**), this did not reach significance (**Figure 5D**).

rhRLX, RGZ, and ISO all significantly inhibited contraction to ET-1 (**Figures 5E–H**), but only rhRLX was effective when these drugs were compared at the same concentration (0.1 μM), where rhRLX reduced contraction to 10^-7^ M ET-1 by 50% (**Figures 5E,H**).

### rhRLX-Mediated Inhibition of Tracheal Contraction is Increased with Time and Dependent on Released Factors

**FIGURE 6:**
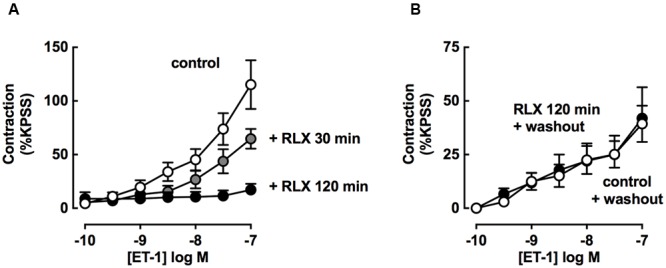
**rhRLX-mediated inhibition of tracheal contraction is increased with time and dependent on released factors.** ET-1-induced contractions were assessed **(A)** in the absence or presence of rhRLX (RLX, 0.1 μM) following pre-treatment with for 30 and 120 min and **(B)** in the absence of RLX following 120 min in the absence or presence of RLX (0.1 μM) followed by replacement of the bathing solution. All responses are expressed as % KPSS standard contraction (mean ± SEM, *n* = 4–7 per group).

The effect of increasing rhRLX preincubation time on the inhibition of development of ET-1 contraction by rhRLX was assessed (**Figure 6A**). Pre-incubation with rhRLX for 120 min completely inhibited the development of ET-1 contraction (*P* < 0.01 vs. control), while 30 min preincubation only partially inhibited contraction.

Trachea were also preincubated in the absence or presence of rhRLX, and contraction to ET-1 assessed after the bathing solution was replaced with buffer without rhRLX (washout).

In the absence of rhRLX preincubation, ET-1 mediated contraction after washout was lower than without washout (**Figure 6B** washout compared to **Figure 6A** control). When pre-treatment with rhRLX was followed by washout, there was no inhibition of contraction to ET-1 (**Figure 6B**).

### rhRLX Acts via Epithelial-Dependent and -Independent Mechanisms

**FIGURE 7:**
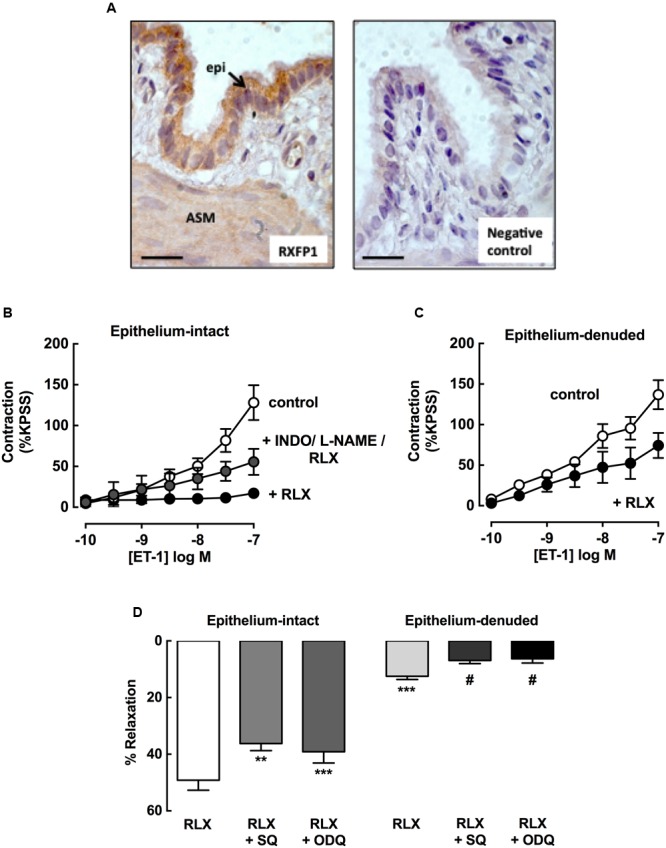
**rhRLX acts via epithelial-dependent and -independent mechanisms.** RXFP1 protein was localized in rat lungs by immunohistochemistry, and responses to rhRLX (RLX) in epithelial-intact and -denuded trachea were compared in the absence and presence of specific inhibitors. **(A)** Immunoreactive RXFP1 is predominantly localized in airway epithelium (epi) and smooth muscle (ASM). Contraction to ET-1 in **(B)** intact trachea under control conditions, and with RLX (0.1 μM) in the absence and presence of INDO (3 μM) + L-NAME (100 μM) and **(C)** in denuded trachea in the absence and presence of RLX (0.1 μM). Responses to ET-1 are expressed as a % of KPSS contraction. **(D)** Responses to RLX (0.1 μM) in intact and denuded trachea in the absence or presence of SQ22536 (10 μM) or ODQ (1 μM), expressed as % relaxation of MCh pre-contraction. Data is expressed as mean ± SEM (*n* = 4–8 per group). ^∗∗^*P* < 0.01, ^∗∗∗^*P* < 0.001, vs. RLX in intact trachea, #*P* < 0.05 vs. RLX in denuded trachea.

To explore potential sites of action of rhRLX, expression of its cognate receptor RXFP1 was determined in rat lungs by immunohistochemistry. RXFP1 was localized in rat airways, with a greater intensity of staining detected in airway epithelium than smooth muscle (**Figure 7A**).

To explore potential mediators of inhibition of contraction by rhRLX, responses to ET-1 were determined in intact trachea after 120 min preincubation with rhRLX in the absence or presence of indomethacin and/or L-NAME. Contraction to 100 nM ET-1 (125 ± 24% KPSS) was almost completely abolished in the presence of rhRLX (17 ± 6% KPSS). Neither inhibitor alone significantly reduced this inhibitory effect of rhRLX (data not shown). However, the marked inhibition of contraction to ET-1 by rhRLX was reduced in the presence of the combination of indomethacin and L-NAME (56 ± 7% KPSS, *P* < 0.01 cf. ET-1+rhRLX alone) (**Figure 7B**). This implicates both COX- and NOS-dependent pathways in the inhibitory effect of rhRLX on contraction.

The tracheal epithelium was then removed in order to assess epithelial-dependence. Denudation was confirmed by conventional hematoxylin staining of formalin-fixed sections (data not shown) and reduced responsiveness to substance P (% relaxation: intact 92.4 ± 2.7%; denuded 10.1 ± 4.0, *P* < 0.01). In contrast to results obtained in intact trachea (control, **Figure 7A**), preincubation with rhRLX for 120 min only partially inhibited contraction to ET-1 following epithelial removal (**Figure 7C**).

The effects of epithelial removal on relaxation to rhRLX following pre-contraction with MCh was also assessed (**Figure 7D**). The level of MCh pre-contraction was similar in epithelial -intact and -denuded trachea (ΔmN: intact 2.4 ± 0.1, denuded 2.2 ± 0.7, NS). Although relaxation to rhRLX (0.1 μM) was reduced by 40% with epithelial removal, a small but significant reversal of the contraction to MCh was still evident (**Figure 7D**). Inhibition of adenylate cyclase and guanylate cyclase, with SQ22536 and ODQ respectively, reduced relaxation to rhRLX in both intact and epithelial-denuded trachea.

## Discussion

This study provides the first evidence that rhRLX is an effective bronchodilator and bronchoprotective agent in rat and guinea pig airways. Compared to the β-adrenoceptor agonist ISO and the novel dilator RGZ, rhRLX induced slower relaxation in precontracted rat trachea, with more rapid relaxation elicited in intrapulmonary airways in rat PCLS. The actions of rhRLX appeared to be mediated by the release of endogenous factors [NO, prostaglandin E_2_ (PGE_2_)], but were only partially epithelial-dependent. rhRLX also markedly increased the potency of β-adrenoceptor agonists ISO and SAL in guinea pig trachea, and potentiated the extent and rate of ISO- and RGZ-mediated relaxation respectively in rat trachea. These data suggest that rhRLX may target alternative dilator mechanisms and offer potential benefit in combination treatments to overcome excessive bronchoconstriction in asthma.

Bronchodilators are the mainstay of pharmacologic therapy for relief of asthma symptoms. However, there is an unmet medical need for more effective treatment under conditions of reduced responsiveness to β-adrenoceptor agonists. Identifying novel bronchodilators that target alternative mechanisms may offer additional benefit during a severe asthma attack or following β-adrenoceptor desensitization (Cazzola et al., 2012).

To date, the acute effects of rhRLX in the regulation of airway contraction have not been examined. Relaxin is a pregnancy-related hormone, but has also been shown to elicit relaxation of rat renal and mesenteric arteries (McGuane et al., 2011; Leo et al., 2014). In the context of the lung, chronic treatment with rhRLX protected against the development of airway remodeling, reversed established fibrosis and reduced AHR in chronic AAD models (Royce et al., 2009, 2013a).

In this study, we assessed potential dilator effects of rhRLX in comparison with the non-selective β-adrenoceptor agonist ISO and the selective β_2_-adrenoceptor agonist SAL. We also compared rhRLX with the PPARγ agonist RGZ, which was recently shown to exert acute relaxation in mouse trachea and small airways (Bourke et al., 2014; Donovan et al., 2014). We utilized MCh, the gold standard to induce airway contraction both *in vitro* and *in vivo* (Donovan et al., 2013), and ET-1, which is upregulated in asthma (Trakada et al., 2000) and contributes to both airway contraction and fibrosis (Ahmedat et al., 2013).

rhRLX was shown to be an effective but slowly acting dilator, reversing an established MCh-induced contraction in both trachea and intrapulmonary airways in PCLS. Of note, the efficacy of the highest concentration of rhRLX tested was similar to the maximal response to ISO, but this partial relaxation to rhRLX in both large and small airways was achieved at a 10-fold lower concentration than the β-adrenoceptor agonist. RGZ was confirmed as a maximally effective dilator of rat airways, with potency in the high μM range consistent with previous data obtained in mice (Donovan et al., 2015). Future studies will apply higher concentrations of rhRLX than those available for this study to see if complete relaxation can be achieved with greater potency and efficacy than β-adrenoceptor agonists.

Pretreatment with rhRLX (0.1 μM) also inhibited the development of tracheal contraction to the potent bronchoconstrictor ET-1. Similar inhibition was seen with effective dilator concentrations of ISO and RGZ (1 and 100 μM respectively), but only rhRLX was effective when comparisons were made at the same concentration and preincubation time (0.1 μM, 30 min). These findings support a greater bronchoprotective action of rhRLX, in addition to its dilator actions, both evident at lower concentrations than ISO and RGZ.

To identify its site of action, we showed that RXFP1, the cognate receptor for rhRLX, was expressed in both airway epithelium and smooth muscle. In addition, inhibition of airway contraction and relaxation to rhRLX was reduced, but not abolished, by epithelial removal. This is in contrast to acute vascular relaxation to rhRLX that was abolished by endothelial removal alone (McGuane et al., 2011) despite localization of RXFP1 in both endothelial and vascular smooth muscle cells (Novak et al., 2006; Jelinic et al., 2014). In the absence of a commercially available RXFP1 antagonist to confirm receptor-dependence, rhRLX appears to be regulating airway contraction via actions on both epithelium and ASM. Further assessment when RXFP1 expression is reduced or abolished, either by using siRNA strategies *ex vivo* or using airway preparations from a knockout model, should be considered to confirm this mechanism of action.

In exploring the mechanisms underlying the effects of rhRLX, it was notable that rhRLX-mediated relaxation was more rapid in small airways than trachea, occurring within minutes under static conditions. In addition, rhRLX-mediated inhibition of tracheal contraction was almost completely abolished with longer pretreatment, and prevented when the bathing solution was removed prior to assessing contraction. Potentially higher levels of endogenous mediators of relaxation released in response to rhRLX would be present in the relatively smaller chamber used for the lung slice studies or with longer time of exposure. While it remains to be confirmed whether the increased rate and extent of relaxation to RGZ and ISO respectively seen in trachea when in combination with rhRLX is also evident when tested in PCLS, these combined findings are consistent with the requirement for the release and accumulation of endogenous factors for relaxation in response to rhRLX.

The potential identity and origin of these endogenous mediators were then explored. In intact trachea, inhibition of contraction by rhRLX was reduced in the presence of the combination of the NOS inhibitor L-NAME or the COX inhibitor indomethacin, and also by epithelial removal. This implicates both PGE_2_ and NO, released from the epithelium, as endogenous mediators contributing to the effects of rhRLX. However, PGE_2_ release from ASM has been shown to exert autocrine effects on proliferation and cytokine release (Holgate et al., 2003). Since inhibitory effects of rhRLX on contraction were still evident in epithelial-denuded tissues, rhRLX may also act via RXFP1 on ASM to increase levels of ASM-derived mediators such as PGE_2_ and/or exert direct effects on ASM to oppose contraction. In recent studies, rhRLX was shown to increase prostacyclin release to exert relaxation in vascular tissue (Jelinic et al., 2014; Leo et al., 2014; Sarwar et al., 2016). This further suggests that prostaglandins are key contributors to rhRLX-mediated relaxation.

The guanylate cyclase inhibitor, ODQ and the adenylate cyclase inhibitor SQ22536, in both intact and epithelial-denuded trachea, reduced relaxation to rhRLX to reverse MCh-induced contraction. This finding is also consistent with contributions of NO and PGE_2_ released from epithelium and/or ASM in response to rhRLX, as these mediators activate GC and AC respectively in intact tissue to oppose ASM contraction. However, rhRLX itself has been shown to directly increase cAMP and activate PKA in both transfected HEK cells expressing RXFP1 (Halls et al., 2007) and bronchial epithelial cells to stimulate migration and ciliary beat frequency (Wyatt et al., 2002). Overall, the mechanisms driving relaxation appear to involve indirect effects of rhRLX mediated by NO, and PGE_2_ and direct effects of rhRLX on both epithelial cells and ASM.

Characterization of the mechanisms and relative efficacy of novel dilators is necessary to support studies of their potential benefit in combination with existing therapy with β-adrenoceptor agonists. We have previously shown that RGZ reversed MCh-induced contractions in mouse trachea and lung slices through PPARγ- and epithelial-independent mechanisms involving the attenuation of Ca^2+^ oscillations (Bourke et al., 2014; Donovan et al., 2015). We assessed the effects of rhRLX in combination with RGZ, ISO and SAL, to determine whether the mechanism of action of rhRLX, newly identified here, would increase the extent or rate of relaxation to RGZ or the β-adrenoceptor agonists.

Although, the combination of RGZ and rhRLX was not more effective at eliciting relaxation in rat trachea, the response was markedly more rapid than either treatment alone. In addition to its epithelial-independent mechanisms, RGZ has also been shown to inhibit PGE_2_ breakdown (Henry et al., 2005), so the PGE_2_-dependent pathways contributing to rhRLX-mediated relaxation may have been further enhanced in this particular combination.

The effects of rhRLX in combination with ISO were tested in rat trachea, where relaxation is mediated via both β_1_- and β_2_-adrenoceptors and ISO elicits rapid but incomplete relaxation. Under these conditions, rhRLX increased ISO-mediated relaxation at a similar rate compared to ISO alone, despite the slow relaxation seen with rhRLX alone. In guinea pig trachea, complete relaxation was mediated via β_2_-adrenoceptor agonism by SAL alone, overcoming the contraction due to MCh and any endogenous contractile mediators that may have accumulated during the experiment. SAL was more potent than ISO, and rhRLX markedly increased the potency of both bronchodilators. Thus, although the rapid accumulation of cAMP in response to ISO or SAL may be maximal, the additional ability of rhRLX to generate cGMP (via NO) may contribute to greater relaxation to β-adrenoceptor agonists when their efficacy is limited as in rat trachea, or increase their potency to further enhance dilator responsiveness as in guinea pig trachea.

rhRLX therefore offers intriguing possibilities as an alternative or additional therapy for asthma. Phase 3 clinical trials are already underway for the use of rhRLX to treat acute heart failure, while its safety and efficacy have been established (RELAX-AHF trial, Teerlink et al., 2013). rhRLX has previously been demonstrated to oppose the development of airway remodeling and AHR, and shown to have greater anti-fibrotic effects when used in combination with prednisolone in an experimental model of AAD (Royce et al., 2013b). Here we demonstrate further potential benefits of rhRLX, mediating bronchodilator actions by mechanisms that differ from and potentially enhance responses to β-adrenoceptor agonists. It would therefore be of interest to assess both its bronchodilator efficacy and potential anti-inflammatory actions in this disease context to support its further preclinical evaluation. Future studies should define the therapeutic potential of Serelaxin as an add-on reliever medication for asthma, particularly when β-adrenoceptor responsiveness is limited.

## Author Contributions

ML designed and performed experiments and assays, and contributed to the writing of the manuscript. CD and MJ performed experiments and contributed to the interpretation of the results and the preparation of the manuscript. SR, LP, and CS contributed to the design of experiments, interpretation of the results and the preparation of the manuscript. JB conceived and organized the study and was the PI, contributed to the design of experiments, interpretation of the results, and wrote the first draft of the manuscript.

## Conflict of Interest Statement

The authors declare that the research was conducted in the absence of any commercial or financial relationships that could be construed as a potential conflict of interest.
